# Assessment of Intraoperative Blood Loss during Oral and Maxillofacial Surgical Procedures in a Nigerian Tertiary Health Care Center

**DOI:** 10.1155/2014/301467

**Published:** 2014-09-02

**Authors:** Babatunde O. Akinbami, Bisola Onajin-Obembe

**Affiliations:** ^1^Department of Oral and Maxillofacial Surgery, University of Port Harcourt Teaching Hospital, Choba, PMB 6173 Port Harcourt, 500004 Rivers State, Nigeria; ^2^Department of Anaesthesia, University of Port Harcourt Teaching Hospital, PMB 6173 Port Harcourt, 500004 Rivers State, Nigeria

## Abstract

*Background*. Reports on estimated amount of blood loss in maxillofacial surgical procedures will guide clinicians through units of blood required for each procedure. The aim of the study was to assess the amount of blood loss and duration of surgery. *Methods*. All cases of maxillofacial surgical procedures done under GA in the MFU theatre, from January 2007 to December 2013, were included in the study. Pre- and postoperative haematocrit values, number of units of whole blood requested and used, amount of blood loss, and duration of surgery were recorded. *Results*. 139 patients were analyzed, of which 75 (54.0%) were males and 64 (46.0%) were females. Fifty-six (40.3%) cases involved soft tissues. Eighty-three cases involved hard tissues. Age range was 2 months to 78 years; mean ± (SD) was 21.3 ± (18.5) years. Isolated unilateral cleft lip had the lowest mean value of estimated blood loss of 10.4 ± 10.8 mLs and also the lowest duration of surgery of 58 (76) minutes. There was no significant relationship between both parameters for cleft lip. Fractures of the mandible had mean blood loss of 352 mLs and duration was 175 min. *Conclusion*. In this study, there was significant relationship between estimated blood loss and duration of surgery for mandibular and zygomatic complex fractures.

## 1. Introduction

Intraoperative blood loss is one of the causes of death during surgical procedures [1]. Acute anaemia can also result from excessive blood loss and this can affect healing of tissues after surgery [1]. Maxillofacial surgical procedures can be classified as minor, intermediate, major, or supramajor cases based on the type and duration of the procedures [2–9]. These procedures may be associated with excessive blood loss from the facial microvasculature and major blood vessels within the operation field of the surgeon [3, 10–14]. Quite often, the lesions have also invaded the walls of the vessels [3] or lie close to these vessels, thereby making them vulnerable to injury during surgery with consequent loss of blood. Furthermore, a significant amount of bleeding can occur during dissection of the capillary-rich skin, subcutaneous tissue, and muscles in the maxillofacial region. Various strategies for preventing excessive blood loss have been applied to maintain haemostasis and these also include the use of hypotensive anaesthesia and tranexamic acid [4].

Patients may be required to donate varying number of units of blood prior to surgery which may or may not be used. The potential blood loss and estimated number of blood products required should therefore be predetermined using many factors [7]. These include haemoglobin or hematocrit levels, body weight of the patient especially for paediatric cases, extent of the lesion, age, and gender as well as the type and extent of procedure. The experience of the surgeon and possibly the duration of surgery must also be considered. Madsen et al. [15] evaluated intraoperative blood loss by thromboelastography (TEG) and they stated that it was an objective method of assessing and predicting intraoperative blood loss.

Much attention has been given to blood loss following general and orthognathic surgeries in the literature, but little work has been done on other maxillofacial procedures [5–9, 15–20]. The aim of the study was therefore to assess the amount of blood loss and the number of units of whole blood required for oral and maxillofacial procedures and also to evaluate any relationship between the amount of blood loss and duration of surgery.

## 2. Methods and Patients

Ethical approval to carry out the study (UPTH/ADM/90/S.II/VOL.X/371) was provided by the University of Port Harcourt Hospital's Ethics and Research Committee (Chairperson Professor Anthony Okpani) on January 27, 2014. All cases of maxillofacial surgical procedures done under GA in the MFU theatre, from January 2007 to December 2013, were included in the study. Patients' demographics and haematological profile retrieved from the case files and theatre records by the house officer and cross-checked by one of the consultants (B. O. A) were documented in a retrospective review chart. Data included the extent, diagnosis of lesion, and medical comorbidities. Pre- and postoperative haematocrit values, number of units of whole blood requested, cross-matched, and used, procedure, amount of blood loss, and duration of surgery were recorded. All cases done under local anaesthesia in the clinic were excluded from the study. The cases were divided into two groups which were diseases or procedures on soft and hard tissues. The total blood loss estimation was done by calculating the amount of blood in the suction bottle and adding this to the estimated value from all the blood soaked gauze.

Data obtained was analyzed with SPSS (SPSS Inc., Chicago, IL) version 16. Means and standard deviation of haematocrit values, estimated blood loss, and duration of surgery for each category of disease were determined and the means within groups and between the two groups were compared with paired sample *t*-test. Linear regression analysis was used to analyze the association between blood loss and duration of surgery. *R* coefficients and significance values were determined. *P* values equal to or less than 0.05 were considered significant.

## 3. Result

A total of 139 patients were analyzed, out of which 75 (54.0%) were males and 64 (46.0%) were females; age range was 2 months to 78 years; mean ± (SD) was 21.3 ± (18.5) years. Fifty-six (40.3%) cases involved soft tissues. Isolated cleft palate 19 (13.7%) and cleft lip 18 (12.9) constituted the highest number of cases seen. Range and mean haematocrit values of soft tissue lesions are reflected in Table 1. Cases of malignant soft tissue tumours presented with the lowest preoperative haematocrit and cases of soft tissue tumours had the highest mean value. Up to 3 units of blood were requested for malignant tumours, but we mostly used 2 units for the less extensive resections. Lowest preoperative haematocrit level taken for elective surgery was 21% and this was in cleft lip.

Eighty-three cases involved hard tissues. Range and mean haematocrit values of bony lesions are reflected in Table 2. Cases with multiple fractures presented with the lowest preoperative haematocrit and cases of cysts/fibroosseous lesions had the highest mean value. Up to 3 units of blood were requested for resection and reconstruction in mandibular tumours, but we mostly used 2 units.

Isolated unilateral cleft lip had the lowest mean value of estimated blood loss of 10.4 ± (10.8) mls and also the lowest duration of surgery of 58 (76) mins. There was no significant relationship between both parameters for cleft lip (Table 3). Comparison of mean values of blood loss and duration between isolated cleft lip and isolated palates gave *P* > 0.05. Complete cleft of primary and secondary palate recorded blood loss of 400 mLs with a mean duration of 4 hrs 23 mins, but the correlation coefficient, 0.327, was not significant, with a *P* > 0.05.

The association between blood loss in benign soft tissue tumours, 360 mls, and duration of surgery, 2 hrs 10 mins, was the least significant, *P* > 0.05. For fractures of the mandible blood loss was 352 mLs and duration was 175 min, significance: *P* < 0.05 (Table 4). Zygomatic complex fractures recorded blood loss of 248 mLs and duration of 185 mins, significance: *P* < 0.05.

In mandibular tumours, blood loss was 1214 mLs and duration was 5 hrs 30 min. There was no relationship between both parameters. In maxillary tumours treated by hemimaxillectomy, mean blood loss was 627 mLs and duration was approximately 2 hrs; the relationship was not significant with *P* value as reflected in Table 4. Comparison of mean values of blood loss and duration between mandibular and maxillary tumours gave *P* > 0.05.

When the mean blood loss in the two groups was compared, there was significant difference, *R* coefficient of 0.935, *P* < 0.05. By comparison of means of duration of surgery, *R* coefficient was 0.817 and *P* < 0.05 also showed significant differences between the two groups.

## 4. Discussion

Intraoperative blood loss can be predicted by preoperative thromboelastography which measures the interaction between coagulation factors, platelets, and fibrinolytic agents. Parameters measured included the clot formation time, maximal clot firmness, fibrinolytic resistance of clot, and *α* angle. Madsen et al. [15] separated their patients into 2 groups based on intraoperative bleeding volume (≤400 mLs and >400 mL); they found no significant associations between routine anticoagulant tests and intraoperative blood loss. When the TEG results for the two groups were compared, there was significant association between clot formation time, maximum clot firmness, and alpha angle, but the fibrinolytic resistance of blood clot was not related to intraoperative blood loss and they concluded that alpha angle greater than 67 degrees was suggestive of blood loss of 400 mLs or less with 95% certainty, but such predictions may not reflect actual values.

Eipe and Ponniah [18] opined that differences in pre- and postoperative haematocrit values and deductions of blood loss by the Gross formula are invaluable; the formula stated that actual blood loss equals blood volume multiplied by the difference in pre- (initial) and postoperative (final) hematocrit values and divided by mean of both hematocrit values; blood volume was calculated by multiplying body weight in kilograms by 70 mL/kg; however, the values are usually difficult to correlate with exact intraoperative loss due to intraoperative blood transfusion and crystalloid infusions as well as postoperative blood losses/fluid dilutions [18]. Hence clinical estimates of intraoperative blood loss are quite useful and this is done before irrigating wounds with any fluid.

In the operating room, considering the controversy surrounding the use of a discrete concentration of haemoglobin as a transfusion trigger for managing acute blood loss, the anaesthetists mainly depend on the clinical estimation of blood loss which includes checking for pallor and the trends of the patient's oxygen saturation, capillary perfusion, blood pressure, and heart rate patterns. Therefore, each patient was assessed individually and blood transfusion was patient-specific.

In this study, the higher blood loss as well as the longer duration of surgery recorded during operations on hard tissues when compared with soft tissues was likely due to the significant amount of blood loss while dissecting the soft tissue overlying bone before resecting the affected bone itself.

Although our result showed that operations for the excision of malignant soft tissue tumours recorded the highest amount of blood loss and the longest duration of surgery on the whole, this was mainly due to the large dimensions and extent of the tumours involved. Revascularization of abnormally proliferating cells and local spread of the lesion also contributed to increasing bleeding episode seen in our patients.

It is not surprising that, in the hard tissue category, the amount of blood loss and duration of surgery were particularly highest for mandibular tumours undergoing resection and reconstruction of the jaw. The association between these primary and secondary outcome variables was quite significant for mandibular and zygomatic complex fractures but not for tumours of the jaws. Treatment of these fractures involves the dissection and detachment of soft tissues and reflection of the mucoperiosteum overlying the bones and these result in appreciable bleeding. Open reduction and internal fixation of these bone segments are actually major surgeries especially when multiple sites are involved and the number of fracture sites will determine the duration of surgery and amount of blood loss. Our findings will serve as baseline studies for comparison with future studies on intraoperative blood loss from surgical management of facial fractures.

Bell et al. [2] in their study on reconstruction of severely atrophic mandible documented a median blood loss of 300 mls with an interquartile range of 150–1100 mls whereas we had an average of 1214 mLs with a range of 300–2800 mLs. While the study carried out by Pogrel et al. [4] documented a longer duration of operation for vascularized bone grafts (VBG), they however documented equal blood loss (1,100 mLs) during reconstruction of mandible with vascularized and nonvascularized bone grafts. The main reason why our patients bled more may be accounted for by the extent of the benign and locally aggressive nature of ameloblastomas of the mandible seen in our environment. Also contributing to the bleeding were the bone grafts harvested from both anterior and posterior iliac crests. These were used for covering large continuity defects. On the contrary, the relatively less bleeding seen in operations involving maxillary tumours may be due to the fact that most of the tumors were removed by intraoral approach. For maxillary tumours, resections were performed, after which the defect was covered with Sofra-Tulle-wrapped gauze while rehabilitation was accomplished with obturators. However, the amount of blood loss during maxillary tumour resection will also depend on the size and extent of the lesions.

We always use infiltration of adrenaline 1 : 200,000 for up to 15 min prior to the wound incision in addition to hypotensive anaesthesia for major surgeries and these contribute to reduction in blood loss. The meta-analysis of Hardwicked et al. has proven that adrenaline infiltration can reduce bleeding during reduction mammoplasties [21] and the outcome, safety, and efficacy do not depend on the extent/size of the lesions or the tissues involved [22]. Experience has shown that a wider nasal floor mucosa repair in the palate causes more bleeding which is further exacerbated by the diffuse and multiple blood supply of the palate when compared to the skin of the lip.

The American College of Physicians [23] recommended that RBC transfusions should be done unit by unit and reassessment should be done between each transfusion. According to Tartter and Barron [24] excessive intraoperative blood transfusion during surgeries for colorectal malignancies, without reevaluating the haemoglobin concentration in between transfusions, resulted in 90% of the unnecessary transfusions. In our center, the anaesthetists habitually request for a few more units of blood than required which may not be used. This is due to the correct assumption that most maxillofacial procedures are associated with excessive blood loss. On the contrary, our evaluation showed that the highest number of units needed during surgeries for extensive malignant lesions was 3 units while reconstructive surgery for benign tumours of the mandible required 2 units of whole blood. Essentially, blood transfusion may be indicated in cases where the preoperative haemoglobin value used as the transfusion trigger was less than 8 mg/dL.

The lowest preoperative packed cell volume taken for elective surgery in our study was 21%. The benefits of performing operations on patients with low PCV or haematocrit values should be weighed against the risks while blood must be made available in case intraoperative transfusion is required. Notwithstanding, the decision to operate despite a low preoperative haematocrit value as in this case was guided by the favourable anticipated amount of blood loss and duration of surgery.

Apart from maintaining haemostasis, care must be taken to prevent excessive blood losses by avoiding major blood vessels. The approach of lesions via avascular planes, as well as subperiosteal dissections for noninvasive lesions, and safety margin sacrifice of tissues in infiltrative lesions are excellent techniques for preventing intraoperative bleeding. Considering that blood transfusion has potential complications [25–29] and that blood is also a limited resource, inappropriate use of blood must be discouraged. Blood wastage can be avoided by paying more attention to the expected blood loss and using preset criteria for homologous blood administration [23, 30, 31].

In maxillofacial patients, allogenic transfusion can be minimized by intraoperative isovolaemic haemodilution [32]. Unfortunately, autologous blood transfusion and intraoperative blood salvage, commonly used for intracavity operations such as abdominal and thoracic operations, may not be technically amenable to maxillofacial surgeries in our centre. The initiatives taken by the National Blood Transfusion Committees and the use of patient blood management guidelines [33], according to Goodnough and Shander [34], have shown that patient's outcome can be improved by evidence-based transfusion practices, minimization of blood loss, and optimization of patient red blood cell mass.

To provide flexibility, as well as avoid the complications and cost of transfusion, the authors prefer the group and save policy rather than the type and cross-match protocol for lesions with expected blood loss of 500 mLs or less. This blood can then be made available and cross-matched for use in case of unexpected high loss [16, 17, 23].

In conclusion, in this study, there was significant relationship between estimated blood loss and duration of surgery for mandibular and zygomatic complex fractures. The number of units of whole blood requested for was a little higher than the blood loss estimates except for malignant soft tissue tumours, multiple fractures, mandible fractures, and TMJ disorders. The decision was based on precaution, considering the fact that blood may not be available if needed. Multiple factors may be responsible for blood loss during maxillofacial operations, but much still has to do with the physiological status and normal clotting mechanisms of the patients, nature of the lesions, and the use of anaesthetic and surgical control measures.

## Figures and Tables

**Table 1:** Age, gender, hematocrit values, and blood requirements of fifty-six (56) patients with 56 procedures on soft tissues.

Diagnosis	Number of procedures	Age range	Male (%)	Female (%)	Range, mean (SD) pre-PCV	Number of units requested	Number of units used	Range, mean (SD) post-PCV
Cleft lip	18	2 months–35 years	9 (6.6)	9 (6.6)	21–35, 31.4 (4.1)	1	0	27–29, 28 (1.4)
Cleft palate	19	10 months–28 years	4 (2.9)	15 (11.0)	28–38, 33.9 (3.6)	1	0	27–30, 28.3 (1.5)
Cleft lip and palate	4	5 months–20 years	2 (1.5)	2 (1.5)	25–35, 30.3 (5.0)	1	0	24–32, 28.7 (4.2)
Soft tissue tumors	9	4 months–33 years	6 (4.4)	3 (2.2)	33–46, 39.5 (9.2)	2	1	23–44, 33.5 (14.8)
Malignant soft tissue tumors	3	26–32 years	2 (1.5)	1 (0.7)	25–32, 28.5 (4.9)	3	3	34–40, 37.0 (4.2)
Palatal salivary gland tumors	3	26–41 years	3 (2.2)	0 (0)	30–35, 32.5 (3.5)	1	0	—
Total	**56**	—	**26 (18.7)**	**30 (21.6)**	—	—	—	—

**Table 2:** Age, gender, hematocrit values, and blood requirements of eighty-three (83) patients with 83 procedures on bony tissues.

Diagnosis	Number of procedures	Age range (years)	Male (%)	Female (%)	Range, mean (SD) pre-PCV	Number of pints requested	Number of pints used	Range Mean (SD) post-PCV
Fractures of the mandible	17	2–65	14 (10.1)	3 (2.2)	31–38, 33.3 (3.3)	1	1	38–44, 41.0 (4.2)
Zygomatic fractures	6	8–38	4 (2.9)	2 (1.5)	21–44, 29.5 (6.0)	1	0	29–32, 30.5 (2.1)
NOE and Le Fort fractures	6	4–27	3 (2.2)	3 (2.2)	28–35, 31.5 (4.9)	0	0	—
Multiple fractures	3	24–35	2 (1.5)	1 (0.8)	20–31, 25.5 (7.8)	2	2	—
Mandible tumors	24	10–78	11 (8.1)	13 (9.6)	25–44 35.5 (5.2)	3	2	25–42, 34.0 (6.2)
Maxillary tumors	16	6–60	9 (6.5)	7 (5.0)	25–36, 34.3 (2.1)	2	1	32–38, 35 (4.2)
Cysts and fibroosseous	6	11–27	3 (2.2)	3 (2.2)	35–38, 36 (1.7)	2	1	28–42, 35 (9.9)
TMJ ankylosis and dislocation	5	11–64	3 (2.2)	2 (1.5)	25–42, 33.5 (12.0)	1	1	30–35, 33 (2.6)
Total	**83**		**49 (36.0)**	**34 (25)**	—	—	—	—

**Table 3:** Estimated blood loss, duration of surgery, and *P* values of fifty-six (56) patients with 56 procedures on soft tissues.

Diagnosis	Treatment	Estimated blood loss Mean (SD) (mls)	Duration of surgery Mean (SD) (mins)	*R* coefficient	Significance level *P* value 0.05
Unilateral cleft lip	Millard's repair	10.4 (10.8)	58 (76)	0.191	0.464
Cleft palate	Palatoplasty	142 (139)	127 (48)	0.081	0.749
Complete cleft lip and palate	Millard's repair/palatoplasty	400 (265)	263 (58)	0.327	0.788
Benign soft tissue tumors	Excision	360 (164)	130 (82)	0.130	0.835
Malignant soft tissue tumors	Excision	1950 (778)	360 (81)	1.000	0.009
Palatal salivary gland tumors	Excision	367 (293)	83 (40)	0.985	0.109

*P* level < 0.05.

**Table 4:** Estimated blood loss and duration of surgery and *P* values of eighty-three (83) patients with procedures on bony tissues.

Diagnosis	Treatment	Estimated blood loss Mean (SD) (mls)	Duration Mean (SD) (mins)	*R* coefficient	Significance level
Fractures of the mandible	ORIF and IMF	352 (351)	175 (75)	0.585	0.014
Zygomatic complex fractures	Zygomatic bone elevation, antral support, and ORIF	248 (185)	185 (112)	0.966	0.034
NOE and Le Fort fractures	Nasal bone reduction, ORIF, canthopexy, and internal suspension	370 (421)	287 (53)	0.873	0.325
Multiple fractures	Zygomatic bone elevation, internal suspension, and IMF	350 (50)	293 (11)	1.000	0.009
Mandible tumors	Resection and reconstruction	1214 (661)	328 (95)	−0.061	0.843
Maxillary tumors	Resection/hemimaxillectomy	627 (471)	127 (61)	0.550	0.125
Cysts and fibroosseous lesions	Enucleation/excision	530 (327)	103 (28)	0.804	0.103
TMJ ankylosis/dislocation	Interposition arthroplasty	550 (303)	210 (82)	0.715	0.285

*P* level < 0.05.
